# Organohalide respiration: retrospective and perspective through bibliometrics

**DOI:** 10.3389/fmicb.2024.1490849

**Published:** 2024-12-24

**Authors:** Hengyi Liao, Xuhao Wang, Xin Wang, Manman Zhang, Yiji Zhang, Siqi Huang, Hongyan Wang, Huijuan Jin, Jingjing Wang, Xiuying Li, Jun Yan, Torsten Schubert, Frank E. Löffler, Yi Yang

**Affiliations:** ^1^Key Laboratory of Pollution Ecology and Environmental Engineering, Institute of Applied Ecology, Chinese Academy of Sciences, Shenyang, Liaoning, China; ^2^University of Chinese Academy of Sciences, Beijing, China; ^3^Shenyang Pharmaceutical University, Shenyang, Liaoning, China; ^4^Viral Ecology and Omics, Institute of Biodiversity, Friedrich Schiller University Jena, Jena, Germany; ^5^Department of Biochemistry & Cellular and Molecular Biology, University of Tennessee, Knoxville, Knoxville, TN, United States; ^6^Department of Civil and Environmental Engineering, University of Tennessee, Knoxville, Knoxville, TN, United States; ^7^Department of Microbiology, University of Tennessee, Knoxville, Knoxville, TN, United States; ^8^Department of Biosystems Engineering and Soil Science, University of Tennessee, Knoxville, Knoxville, TN, United States; ^9^CAS Key Laboratory of Forest Ecology and Silviculture, Institute of Applied Ecology, Chinese Academy of Sciences, Shenyang, Liaoning, China

**Keywords:** organohalide-respiring bacteria, bibliometric analysis, *Dehalococcoides*, reductive dechlorination, biodegradation, anaerobic bacteria

## Abstract

Organohalide-respiring bacteria (OHRB) play a pivotal role in the transformation of organohalogens in diverse environments. This bibliometric analysis provides a timely overview of OHRB research trends and identifies knowledge gaps. Publication numbers have steadily increased since the process was discovered in 1982, with fluctuations in total citations and average citations per publication. The past decade witnessed a peak in publications, underscoring heightened research activity and extensive collaboration. Thematic analysis identified two primary research foci: mechanistic exploration of OHRB and their interplay with environmental factors. Future research should prioritize elucidating the roles OHRB’s play in biogeochemical cycling, utilizing synthetic biology tools for enhanced biotransformation, deciphering OHRB’s ecological interactions, unraveling their evolutionary pathways, and investigating dehalogenation capabilities in other microorganisms, including archaea. These research directions promise to advance our understanding of microbially-driven organohalide transformations, microbial ecology, and genetic engineering potential, ultimately informing natural organohalide cycling and environmental management strategies.

## 1 Introduction

The extensive industrial and agricultural use of organohalides (i.e., organic compounds containing the halogen elements fluorine, chlorine, bromine, and/or iodine) presents a severe environmental and human health hazard. Their toxicity, potential mishandling, and uncontrolled release endanger ecosystems and human populations (Doherty, 2000; Girones et al., 2021; He et al., 2021; Potapowicz et al., 2020). Growing evidence implicates organohalides as significant contributors to global geochemical carbon and halogen cycles (Atashgahi et al., 2018b; Yang et al., 2020a). Therefore, the biotransformation of organohalides has become an important topic for environmental scientists and microbiologists worldwide. A key breakthrough has been the discovery of organohalide-respiring bacteria (OHRB). These microorganisms possess the unique ability to utilize organohalides as electron acceptors for energy generation under anoxic conditions (Suflita et al., 1982; Holliger et al., 1993; Atashgahi et al., 2016; Jugder et al., 2016). OHRB are widespread across diverse environments, including marine sediments, soils, and freshwater ecosystems (Adrian and Löffler, 2016), where they contribute to the degradation of naturally occurring organohalides derived from various sources, such as marine algae, fungi, and plants (Gribble, 2010, 2023). The activities of OHRB are important for maintaining the balance of organohalides in the environments, as these compounds can have significant impacts on atmospheric chemistry, climate, and ecosystem functioning. By breaking down organohalides, OHRB help mitigate the potential adverse effects of these compounds and facilitate their recycling within biogeochemical cycles. In addition to their ecological significance, OHRB are also highly relevant for bioremediation applications. By harnessing their inherited ability to dehalogenate and detoxify synthetic organohalides, OHRB can be employed to remediate contaminated sites, effectively reducing the environmental and health risks posed by these hazardous compounds. Their utilization in bioremediation strategies represents a sustainable, cost-effective and environmentally friendly approach for *in-situ* remediation of organohalide pollution (Atashgahi et al., 2016; Atashgahi et al., 2018a; Kueper et al., 2014; Stroo, 2010, Stroo et al., 2013; Wang et al., 2018).

Extensive research has been conducted on OHRB, particularly within the realms of chlorinated solvents and pesticides degradation. These investigations encompass a broad spectrum, spanning from practical field applications of OHRB at contaminated sites to fundamental research exploring OHRB physiology and ecology, isolation techniques, and the integration of advanced molecular biology and bioinformatics tools (Chen et al., 2018; Qiao et al., 2022; Xu and He, 2022; Zhao et al., 2020; Gadkari et al., 2018; Ismaeil et al., 2024; Löffler et al., 2013; Rossi et al., 2012; Willemin et al., 2020; Wu et al., 2023). Advanced techniques such as high-throughput amplicon sequencing, metagenomics, and proteomics, are increasingly employed to gain deeper insights into the complex roles and mechanisms of OHRB (Dang et al., 2023; Peng et al., 2020; Qiu et al., 2020). The first evidence for OHRB appeared in a Science paper by Suflita et al. (1982), followed by the identification and isolation of dehalogenating bacteria in the 1990s (Maymó-Gatell et al., 1997; McCarty, 1997) and 2000s (He et al., 2003); the elucidation of the structure and mechanisms of dehalogenase enzymes in 2014 (Bommer et al., 2014) and 2015 (Payne et al., 2015) further marked significant breakthroughs. Despite major progress in OHRB research, challenges remain. The complexities associated with cultivating and isolating OHRB impede the comprehensive understanding of their ecological functions, evolutionary traits, and practical applicability. Consequently, there exists a pressing necessity for continued investigations to elucidate the diversities, ecology and evolution of OHRB (Yang et al., 2020a). An extensive body of scientific literature on OHRB exists, which provides the opportunity for systematic analysis and statistical evaluation to gain comprehensive insights.

Bibliometrics, a quantitative approach for analyzing published literature, offers valuable insights into trends and potential knowledge gaps (Borgman and Furner, 2002; Taddeo et al., 2019; Gao et al., 2023). Despite its wide applications, no bibliometric studies have yet been conducted on OHRB literature. To address this gap, this study employs bibliometric methods to analyze scientific information from the Web of Science Core Collection (WoSCC) spanning from 1988 to 2023, with the aim of comprehensively examining the global research landscape of OHRB and providing guidance for future research. The analysis encompasses a thorough examination of various bibliometric indicators, including publication quantity and citation frequency, research areas, publication outlets, authorship patterns, contributing countries/regions and institutions, and keywords co-occurrence. By synthesizing these findings, the analysis explores the challenges and promising prospects of OHRB research. Ultimately, the study seeks to provide a comprehensive overview of the historical development, current state, and future potential of OHRB research through a bibliometric lens.

## 2 Materials and methods

### 2.1 Data sources

The Web of Science (WoS) Core Collection (WoSCC) database of Clarivate Analytics not only encompasses a broad range of citation index records but also includes numerous influential high-quality journals from around the world, making it one of the primary sources for bibliometric analyses (Chen et al., 2021; Ekundayo and Okoh, 2018; Qin et al., 2024; You et al., 2021; Zhang et al., 2020). To ensure the reliability of the conclusion, this study focused on OHRB and employed the WoSCC database as the data source following the established methods (Donthu et al., 2021; Öztürk et al., 2024). An advanced search strategy was conducted utilizing the following retrieval formula: (TS = (“reductive dehalogenase” OR “dehalogenation” OR “dechlorination” OR “reductive dehalogenation” OR “reductive dechlorination”) AND TS = (“*Sulfurospirillum*” OR “*Shewanella*” OR “*Geobacter*” OR “*Trichlorobacter*” OR “*Desulfovibrio*” OR “*Desulfoluna*” OR “*Anaeromyxobacter*” OR “*Desulfomonile*” OR “*Desulfuromonas*” OR “*Desulfitobacterium*”)) OR TS = (“organohalide-respiring bacteria” OR “*Dehalobacter*” OR “*Dehalococcoides*” OR “*Dehalogenimonas*” OR “*Dehalobium*”). The search encompassed all citation indexes, focused on publications in English, and included both research and review articles. Considering the potential bias caused by the rapid updates in the WoSCC database, literature retrieval related to the OHRB study was completed within 24 hours (Qin et al., 2024). The retrieval date was March 12, 2024. Considering that it is not feasible to collect and compile publication data beyond 2024, including a complete year of publications from that year onwards, all publications published after January 1, 2024 have been excluded (Sun et al., 2022). A total of 1,591 publications were obtained in this search, spanning from 1988 to 2023. All retrieved publications were exported in the format of “Full Records and Cited References” as plain text files for further bibliometric and visual analysis. While we conducted preliminary analyses using PubMed and Scopus to confirm overall trends, we focused our primary analysis on the WoSCC database. WoSCC offers the comprehensive and standardized dataset for bibliometric analysis in the natural sciences, ensuring the robustness and reliability of our findings.

### 2.2 Bibliometric analysis

This study systematically categorized 1,591 publications based on the Web of Science research areas (WoS research areas) assigned by Clarivate Analytics (Zhang and Chen, 2020). Employing both the Bibliometric software package (R language environment) (Aria and Cuccurullo, 2017) and Excel, statistical analyses were conducted, encompassing aspects such as the number of publications, local impact (attributed to various authors, countries/regions, and institutions), Keywords Plus terms, among other relevant parameters. Local impact refers to the influence of the articles/journals/participating authors/countries/institutions within the analyzed dataset (measured by total number of citations, h-Index, g-index and m-index), often used to evaluate the status of articles, authors, countries, and institutions within a specific research field. The detailed explanations of the h-index, g-index, m-index, and additional terminology are presented in Table 1. Briefly, global influence refers to the impact on the entire retrieval database (WoS) used. To enhance clarity and precision, a process of appropriately consolidating full names, abbreviations, and synonyms of authors and research institutions was undertaken. TBtools and software packages (ggplot2, pheatmap, fmsb, and circlize) within the R language environment were then utilized for the visual representation of WoS research areas, publication quantity, local impact, and other pertinent variables. The software VOSviewer (van Eck and Waltman, 2010) was also utilized to examine the collaboration network among authors and institutions, the co-occurrence network of keywords, and to generate a visually intuitive knowledge graph. Further refinement and aesthetic adjustments were executed using Pajek software (de Nooy et al., 2011). This comprehensive analysis delved into the performance metrics of publications, WoS research areas, relevant journals, the most locally cited publications, pertinent authors, countries/regions, affiliations, and Keywords Plus terms.

**Table 1:** Terminology and definitions.

Terms	Definitions	Abbreviation
Local impact	Influence within the analyzed data, measured by the total number of citations, h-index, g-index and m-index.	
Total local citations	The total number of citations from other publications within the analyzed data.	TLC
Mean total local citations per publication	The ratio of the total number of citations from other publications within the analyzed data to the total number of publications.	MTLCPP
Cumulative number of publications	The total number of publications published before a specific point in time.	CNP
Annual number of publications	The total number of publications within a specific year.	ANP
h-index	A scientist (journal) has index h if h of his or her (its) N_p_ papers have at least h citations each and the other (N_p_ – h) papers have fewer than ≤h citations each (Hirsch, 2005).	
g-index	The highest number g of papers that together received *g*^2^ or more citations (Egghe, 2006).	
m-index	The median number of citations received by papers in the Hirsch core (this is the papers ranking smaller than or equal to h) (Bornmann et al., 2008).	

## 3 Results and discussion

### 3.1 Publications performance

**Figure 1:**
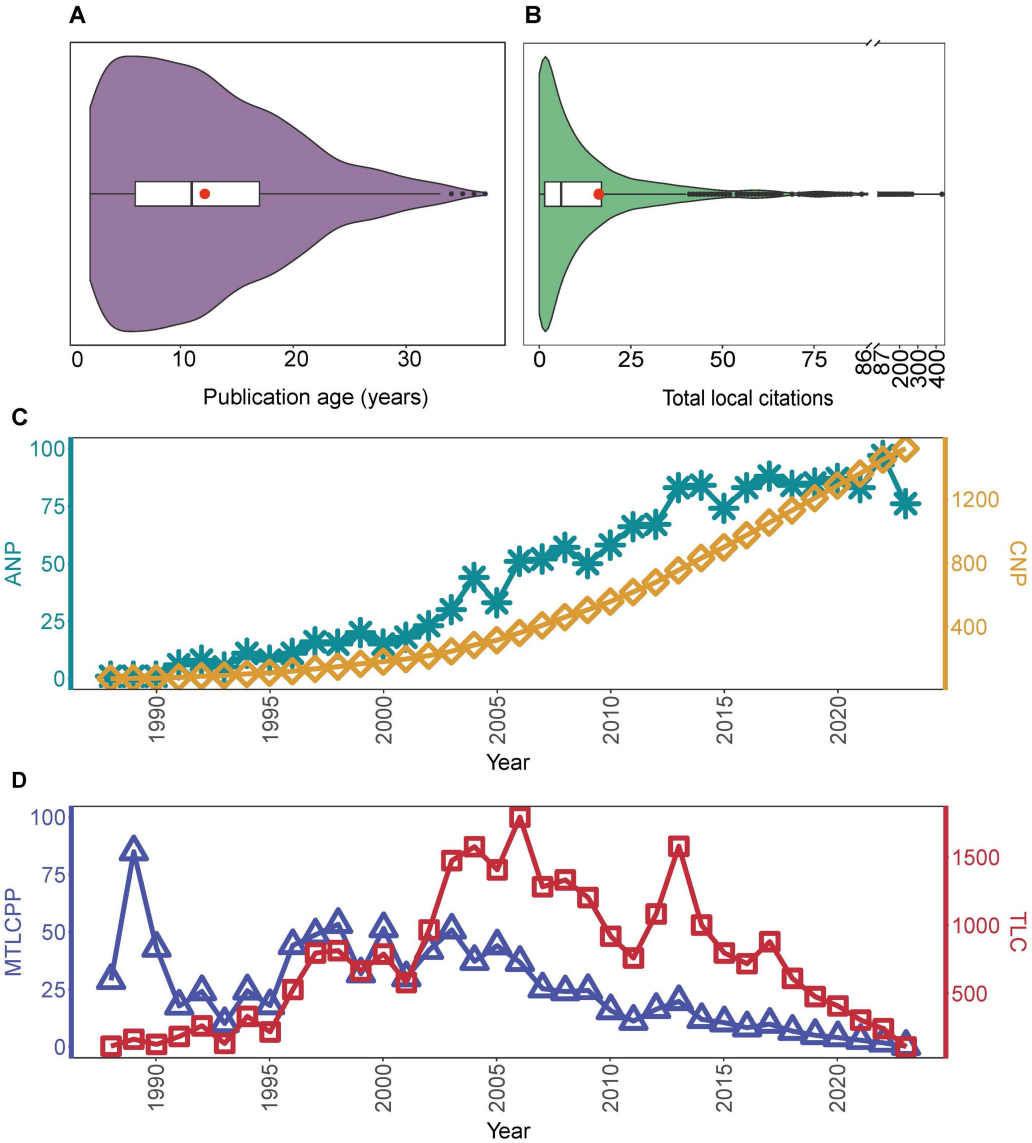
Overview of publications performance from 1988 to 2023. **(A)** The publication age distribution of all publications. **(B)** Total local citations distribution of all publications. **(C)** Cumulative number of publications (CNP) and annual number of publications (ANP) over time. **(D)** Annual publications’ total local citations (TLC) and mean total local citations per publication (MTLCPP) over time. Figures 1A,B, 3C, 4C and 5B are presented as violin plots. Key features include: (1) The shape of the violin body illustrates the data distribution. The width at any given point along the vertical axis corresponds to the density of data points at that value: wider sections indicate higher data density, while narrower sections represent lower density. (2) A box plot is embedded within each violin plot to show key statistical measures: the black bar represents the median value of all publications, and the red dot signifies the mean (average) value. (3) Data points falling outside the expected range are identified as outliers and marked by black dots. Since the annual number of publications in the year 1989 was limited to the Freedman and Gossett paper, this single study substantially influenced the MTLCPP value, resulting in a skewed outcome.

A comprehensive search and screening process within the WoSCC database yielded a total of 1,591 publications related to OHRB. Of these, 1,508 were classified as articles and 83 as reviews. The retrieved publications spanned from 1988 to 2023, with an average publication age of 12.16 years (Figure 1A). The total local citations (TLC), representing the number of citations from other publications within the analyzed dataset, amounted to 25,878. This translates to a mean total local citations per publication (MTLCPP) of 16.27 (Figure 1B). The study of OHRB has witnessed participation from 4,025 authors and 756 research institutions in 50 countries/regions. Figure 1C depicts a significant upward trend in the cumulative number of publications (CNP) within the past 36 years. While the annual number of publications (ANP) exhibits minor fluctuations, the figure displays a steady overall growth and reached its highest value in 2022 (Figure 1C and Supplementary Table S1). Both TLC and MTLCPP of the annual publications demonstrate fluctuating patterns, with their respective peaks occurring in 2006 and 1989 (Figure 1D). While the study of OHRB has witnessed a steady growth in the cumulative number of publications over the past 36 years, reflecting a sustained interest in this field, the declining trend in both total local citations and mean total local citations per publication over time suggests a potential shift in research focus or priorities. This observation could be attributed to factors such as changing funding landscapes, the emergence of new research areas, a shift in research focus, or a decrease in the novelty of findings within the field. For instance, the U.S. Department of Defense (DoD) has historically supported research on reductive dehalogenation through programs like the Environmental Security Technology Certification Program (ESTCP) and the Strategic Environmental Research and Development Program (SERDP)^1^. However, recent funding priorities have shifted towards addressing per- and polyfluoroalkyl substances (PFAS). This shift has influenced the research community to focus on developing and improving PFAS degradation technologies.

### 3.2 Research areas

**Figure 2:**
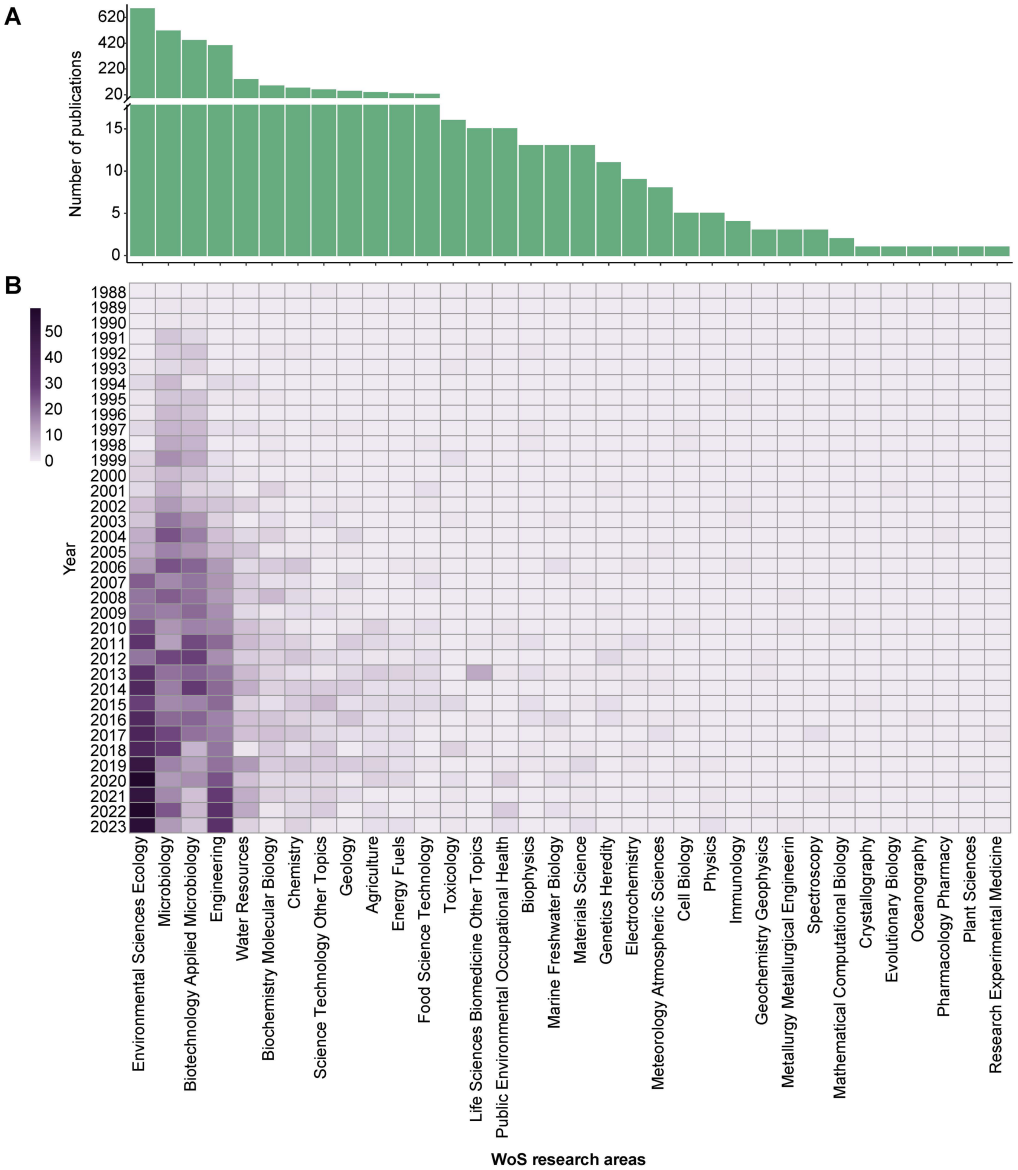
Overview of WoS research areas distribution. **(A)** Number of publications in different WoS research areas. **(B)** Annual publications over time in WoS research areas.

A bibliometric analysis using the WoS research areas classification system provided by Clarivate Analytics examined the distribution of publications across various disciplines within the field of OHRB research from 1988 to 2023 (Figure 2A). The leading research areas encompass Environmental Sciences Ecology, Microbiology, Biotechnology Applied Microbiology, and Engineering. Notably, Environmental Sciences Ecology held the most significant share with 43.18% (*n* = 687) of publications, followed by Microbiology (32.31%, *n* = 514), Biotechnology Applied Microbiology (27.66%, *n* = 440), and Engineering (25.14%, *n* = 400). This observation underscores the critical role played by these core disciplines in shaping the current research landscape of OHRB research. Despite the absolute dominance in terms of publication quantity in the aforementioned leading research fields, OHRB research has also made significant progress in other domains such as Water Resources, Biochemistry Molecular Biology, Chemistry, Geology and Agriculture in recent years (Figure 2B). The analysis further emphasizes the increasing recognition of the importance of interdisciplinary collaboration and cross-disciplinary research in bridging the gap between fundamental research and practical applications within the field. This is particularly relevant in the context of extending *in-situ* bioremediation of organochloride-contaminated sites from the realm of basic research to on-site implementation and practical use. Therefore, fostering and accelerating interdisciplinary cooperation within the field of OHRB research remains a crucial objective for advancing knowledge and achieving successful real-world applications.

### 3.3 Publication landscape and impact

**Figure 3:**
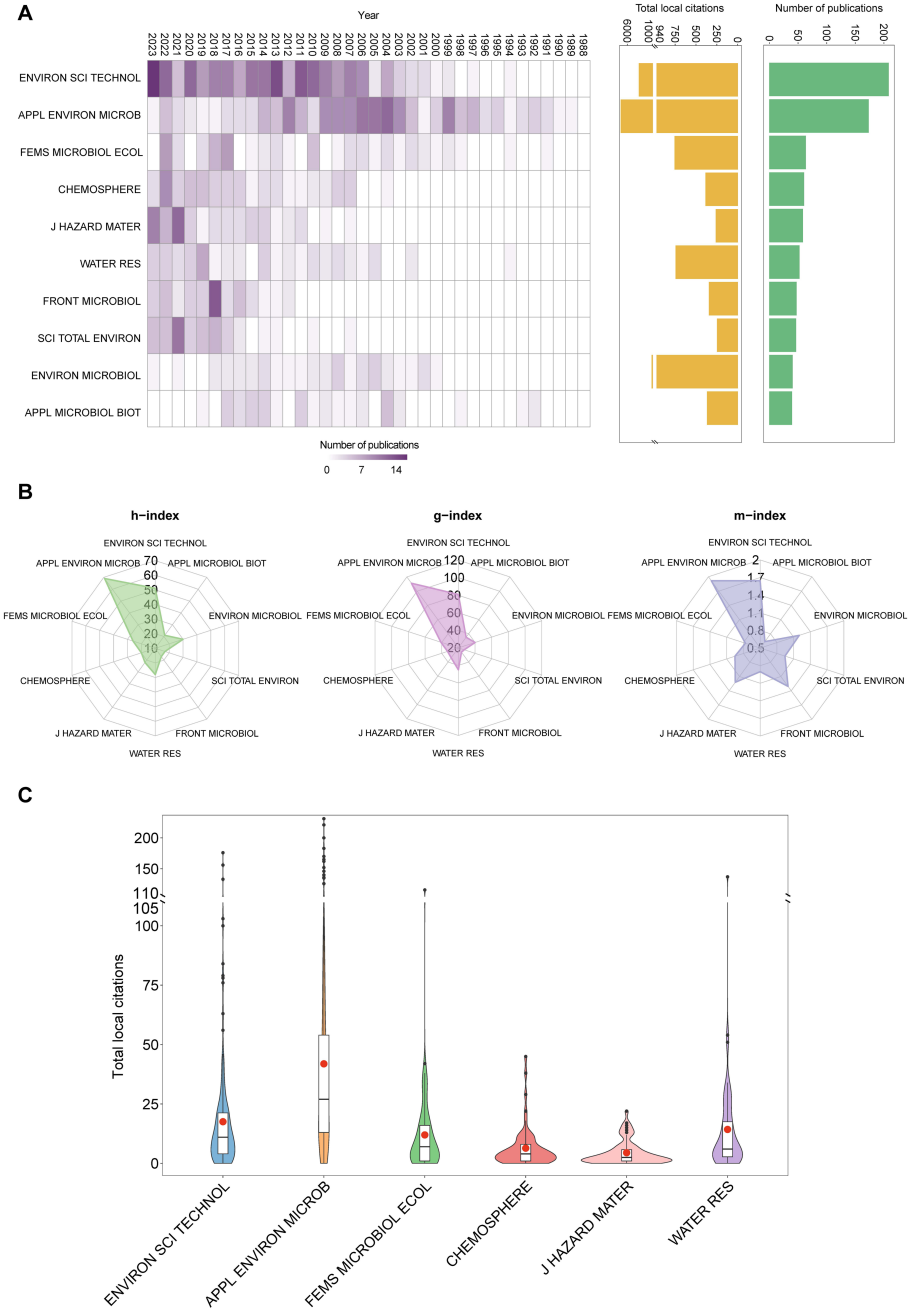
Overview of journals’ publications. **(A)** Top 10 journals publishing research on OHRB showing annual publications over time, total local citations and number of publications. **(B)** Top 10 journals’ h-index, g-index and m-index. **(C)** Total local citations distribution of all documents from the top six journals.

A comprehensive analysis of the publication landscape revealed that between 1988 and 2023, a total of 242 journals published articles related to OHRB research. However, only two journals, Environmental Science and Technology (*n* = 208) and Applied and Environmental Microbiology (n = 173), accounted for a significant portion of the total publications, exceeding 100 articles each. While Environmental Science and Technology held the lead in terms of publication quantity, Applied and Environmental Microbiology emerged as as the leading journal in terms of local impact (Figures 3A,B). This is evidenced by this journal’s superior performance across several bibliometric indicators, including the highest total local citations (7,251), local h-index (69), g-index (111), and m-index (1.917) (Figure 3B). Additionally, the mean total local citations per publication for Applied and Environmental Microbiology (41.91) significantly surpassed those of other journals (Figure 3C). This observed disparity in local impact might be partially attributed to the temporal distribution of publications across these journals. Before 2009, Applied and Environmental Microbiology was the leading journal for OHRB research publications. However, a notable decrease in the number of publications in Applied and Environmental Microbiology has been observed over the last decade. Concurrently, Environmental Science and Technology has gained prominence after 2009, likely due to a growing focus on applying fundamental research findings to develop *in situ* bioremediation technologies. This trend aligns with the increasing interest in translating laboratory discoveries into practical environmental solutions. This difference in publication timelines could explain the lower local impact of the latter journal, as local citations typically accumulate over a longer period. Table 2 presents the top 10 publications with the highest number of local citations. The most influential publication, based on local citations, is titled “Isolation of a bacterium that reductively dechlorinates tetrachloroethene to ethene” by Maymó-Gatell et al. (1997), published in the journal Science. The authors described the first *Dehalococcoides* isolate that dechlorinates PCE to VC and non-toxic ethene (Maymó-Gatell et al., 1997). The second most influential publication detailed strain BAV1, which derives energy from the dechlorination of *c*DCE and VC to ethene (He et al., 2003). Meanwhile, the third most influential publication holds significant importance for establishing the obligate OHRB genus *Dehalococcoides* (Löffler et al., 2013).

**Table 2:** Top ten publications (sorted by the number of local citations).

Rank	Paper	Local citations
1	Maymó-Gatell X, 1997, *Science*	432
2	He JZ, 2003, *Nature*	268
3	Löffler FE, 2013, *Int J Syst Evol Micr*	251
4	Seshadri R, 2005, *Science*	239
5	Müller JA, 2004, *Appl Environ Microb*	231
6	Hendrickson ER, 2002, *Appl Environ Microb*	221
7	Adrian L, 2000, *Nature*	218
8	Holliger C, 1998, *Arch Microbiol*	203
9	Magnuson JK, 2000, *Appl Environ Microb*	200
10	Smidt H, 2004, *Annu Rev Microbiol*	186

### 3.4 Top authors

**Figure 4:**
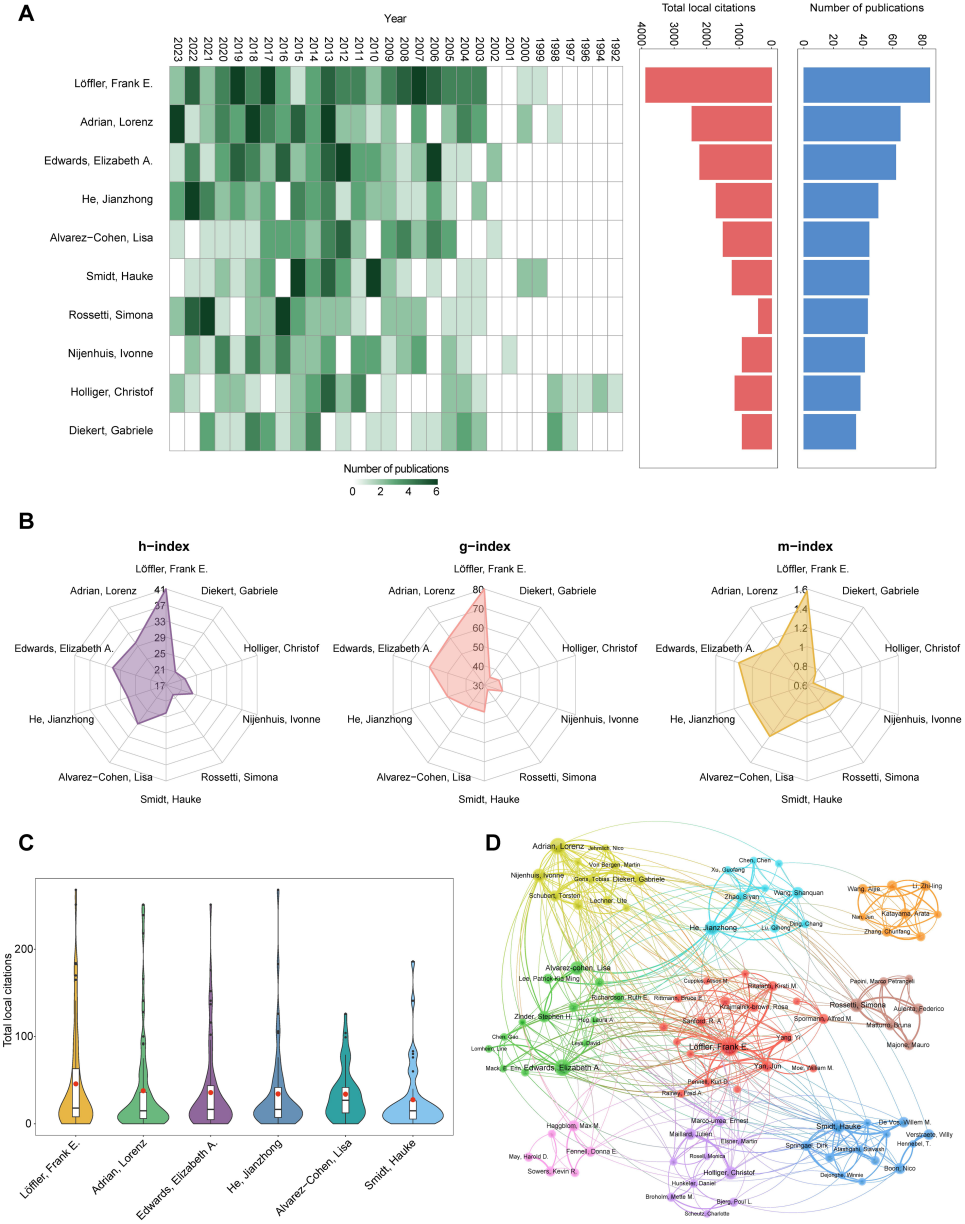
Overview of authors’ publications and collaborations. **(A)** Top 10 (sorted by the number of publications) authors’ annual publications over time, total local citations and number of publications. **(B)** Top 10 (sorted by the number of publications) authors’ h-Index, g-index and m-index. **(C)** Total local citations distribution of all publications from the top six (sorted by the number of publications) authors. **(D)** Main authors’ collaboration network. Each nodes indicates the graph represents one author. The thickness of the connections between nodes is the strength of collaboration between authors.

A bibliometric analysis utilizing the “Authors” feature bar within the Bibliometrix software package (R language environment) identified a total of 4,025 authors contributing to OHRB research during the period 1988–2023. Among the contributing authors, Löffler, Frank (*n* = 85), Adrian, Lorenz (*n* = 65), Edwards, Elizabeth (*n* = 62), He, Jianzhong (*n* = 50), Alvarez-Cohen, Lisa (*n* = 44), Smidt, Hauke (*n* = 44), and Rossetti, Simona (*n* = 43) demonstrated notable productivity, as evidenced by their considerable publication output (Figure 4A). Frank E. Löffler has made significant contributions to the field, as evidenced by a substantial publication record and notable bibliometric indicators (Figure 4B), which include an h-index of 41, a g-index of 80, an m-index of 1.577, a total of 3,878 local citations, and an average of 45.62 local citations per publication. Figure 4C presents a visual comparison of the mean total local citations per publication for these leading authors. Figure 4A reveals a notable upward trend in publications by key authors since 2003, particularly evident between 2012 and 2022, with substantial contributions from authors like Frank Löffler, Lorenz Adrian, Elizabeth Edwards, and others. Although 2023 saw a light dip in output from these authors, research activity in the field is likely ongoing. While it is impossible to individually acknowledge every pioneer and prominent researcher, or to fully evaluate the contributions of all community members solely through numerical metrics, those contributors and authors in the “Yellow Bible” (Häggblom and Bossert, 2003) and “Blue Bible” (Adrian and Löffler, 2016) represent key figures whose work has undeniably shaped the field. The author collaboration network generated by VOSviewer software (Figure 4D) signifies a relatively cohesive network among OHRB researchers. Collaboration appears particularly strong within groups centered around Frank Löffler, Lorenz Adrian, Elizabeth Edwards, Jianzhong He, and others, while connections between these groups and those centered around other major authors seem weak. The observed decrease in publications may be linked to funding trends in Europe and North America, where funding opportunities for OHRB research may have become more limited. For example, the research unit FOR1530 “Anaerobic Dehalogenation: Organisms, Biogeochemistry and (Eco-)Physiology”^2^, funded by the German Research Council (DFG), played a significant role in advancing OHRB research. This initiative fostered the development of prominent researchers and facilitated international collaboration through conferences and workshops. However, the conclusion of this funding program may have impacted the continuity of research and international cooperation.

### 3.5 Countries/regions

**Figure 5:**
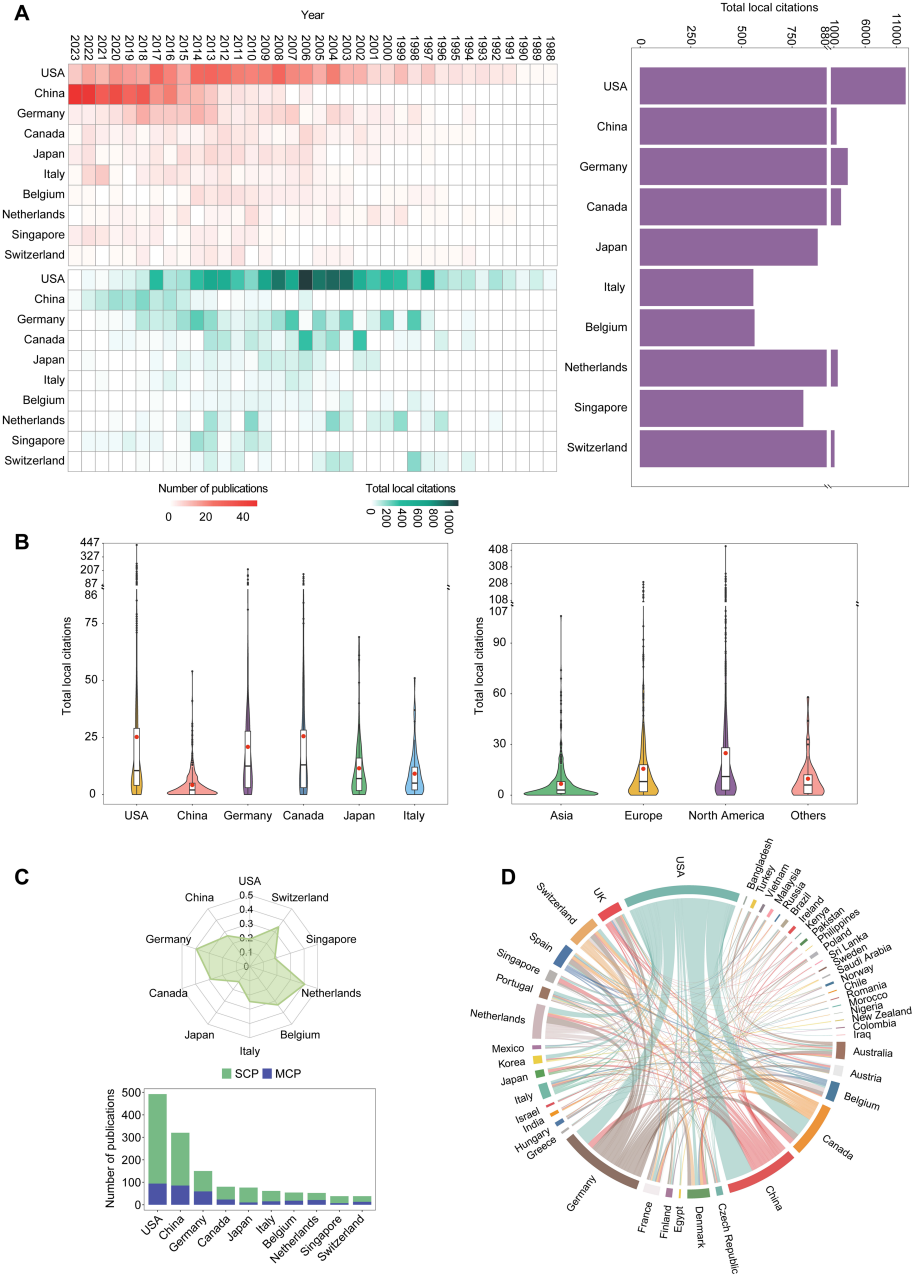
Overview of countries/regions’ publications and collaborations. **(A)** Top 10 (sorted by the number of publications) countries/regions’ annual publications over time, total local citations of annual publications over time and total local citations. **(B)** Total local citations distribution of all publications from the top six (sorted by the number of publications) countries/regions and different continents. **(C)** Top 10 (sorted by the number of publications) countries/regions’ number of publications, international collaboration and MCP ratio. **(D)** Countries/regions’ collaboration network. Each node represents one country/region. The thickness of the connections between nodes indicates the strength of cooperation between countries/regions.

A bibliometric analysis utilizing the “Countries” feature bar within the Bibliometrix software revealed the participation of 50 countries/regions in OHRB research from 1988 to 2023, based on the authors’ work locations. From the perspective of the country or region where the corresponding authors work, the United States (*n* = 492) led the global publication output, followed by China (n = 320) and Germany (n = 150) (Figure 5A). While the United States held the top position in publication quantity, Figure 5A further highlights the disparity in local citations among leading nations. The United States shows a significantly higher total local citation count (12,409) compared to Germany (3,132) and China (1,337). This disparity extends to the mean total local citations per publication, with the United States (25.22) and Germany (20.88) showcasing superior performance compared to China (4.18) (Figure 5B). Despite China’s growing research contributions evident from its publication quantity, its local citation metrics remain comparatively lower. This phenomenon, depicted in Figure 5B, can be attributed to the presence of a substantial number of low-citation publications. The significant rise in China’s publication output since 2013 (Figure 5A) likely plays a role, as citations for newly published works typically accumulate over time (Chen et al., 2023). Notably, Figure 5B reveals a potential regional disparity in local citations, with publications from Asia generally receiving fewer citations compared to those from Europe and North America. Analysis of the international collaboration network using VOSviewer software (Figure 5D) identifies the United States, China, and Germany, the leading countries/regions in publication quantity, forming the core of global cooperation in OHRB research. Despite China’s substantial publication output, its level of international collaboration is slightly lower compared to Germany. As highlighted by Pei et al. (2022), the proportion of publications co-authored with international collaborators (Multiple Country Publications - MCP) relative to the total publications (Single Country Publications - SCP) can serve as another indicator of international cooperation intensity (Pei et al., 2022). Figure 5C demonstrates that Germany, with a lower publication quantity than the United States and China, exhibits a higher international co-authorship rate (MCP ratio). These observations suggest that while China continues to increase its output in OHRB research (Figure 5A), fostering stronger international collaborations could be a crucial step in enhancing research impact and global recognition within this field.

### 3.6 Institutional affiliations and collaborations

**Figure 6:**
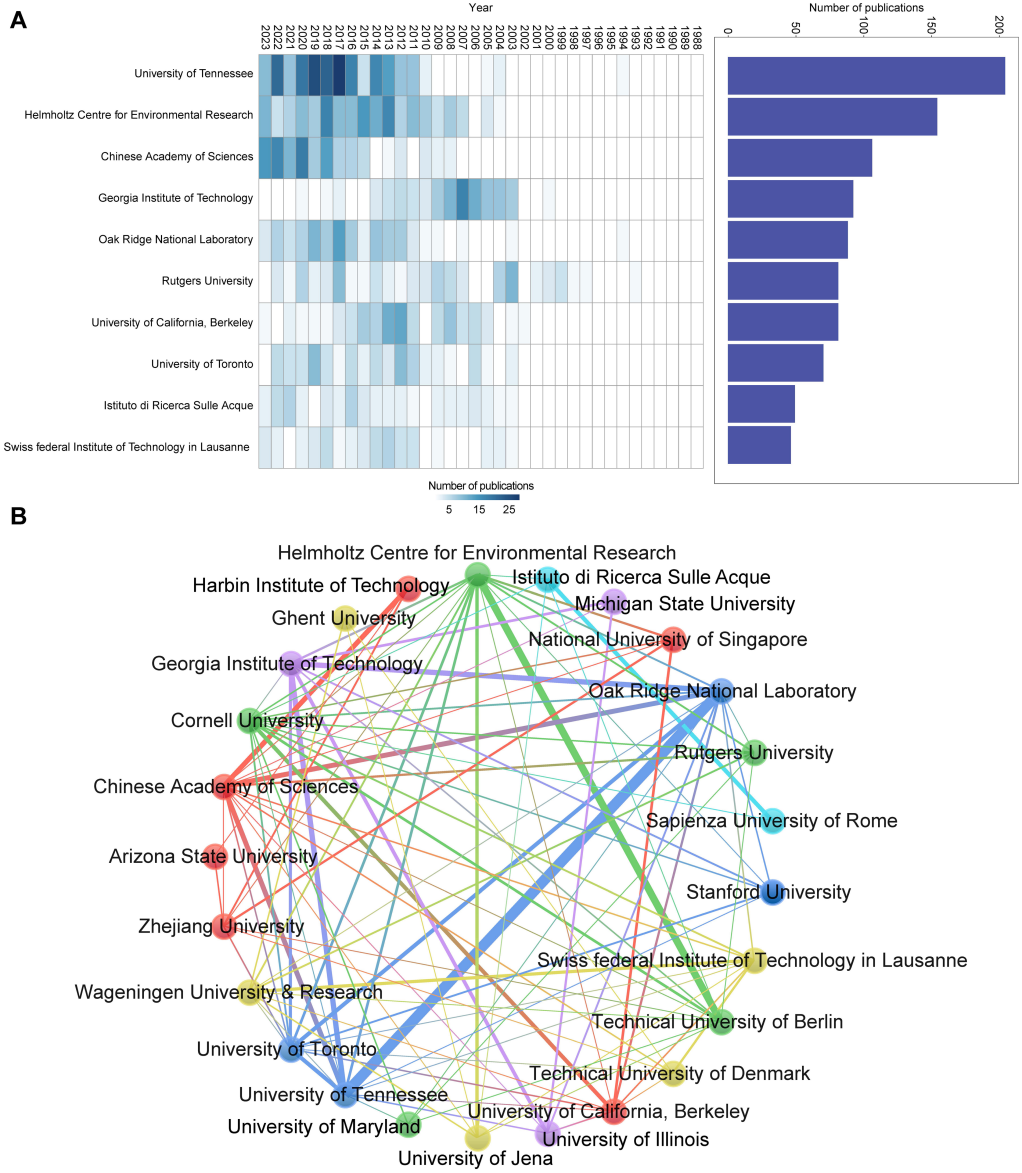
Overview of institutions’ publications and collaborations. **(A)** Top 10 (sorted by the number of publications) institutions’ annual publications over time. **(B)** Main institutions’ collaboration network. Each node represents one institution. The thickness of the connections between nodes indicates the strength of cooperation between institutions.

Bibliometric analysis of the “Associations” feature within the Bibliometrix software reveals the involvement of 756 research institutions in OHRB research between 1988 and 2023. This analysis underscores the longstanding dominance of the United States in this field, with a preponderance of North American institutions leading the publication quantity rankings. This finding demonstrates consistency with the previously observed national distribution of publications. The University of Tennessee in the United States has been a major contributor, with involvement in a substantial number of publications (Figure 6A). The Helmholtz Centre for Environmental Research (UFZ) in Germany has also made significant contributions. The Chinese Academy of Sciences, while a more recent entrant in OHRB research, has demonstrated a rapid increase in publication output (Figure 6A) and actively collaborates with various institutions. However, its collaborative ties with leading institutions in the United States and Germany appear less noticeable. The analysis using VOSviewer software (Figure 6B) reveals strong collaborative networks among key institutions. These findings underscore the United States’ historical and continued prominence in OHRB research, while also highlighting the importance of both independent research and robust inter-institutional collaboration for the field’s advancement.

### 3.7 Keywords analysis and research hotspots

**Figure 7:**
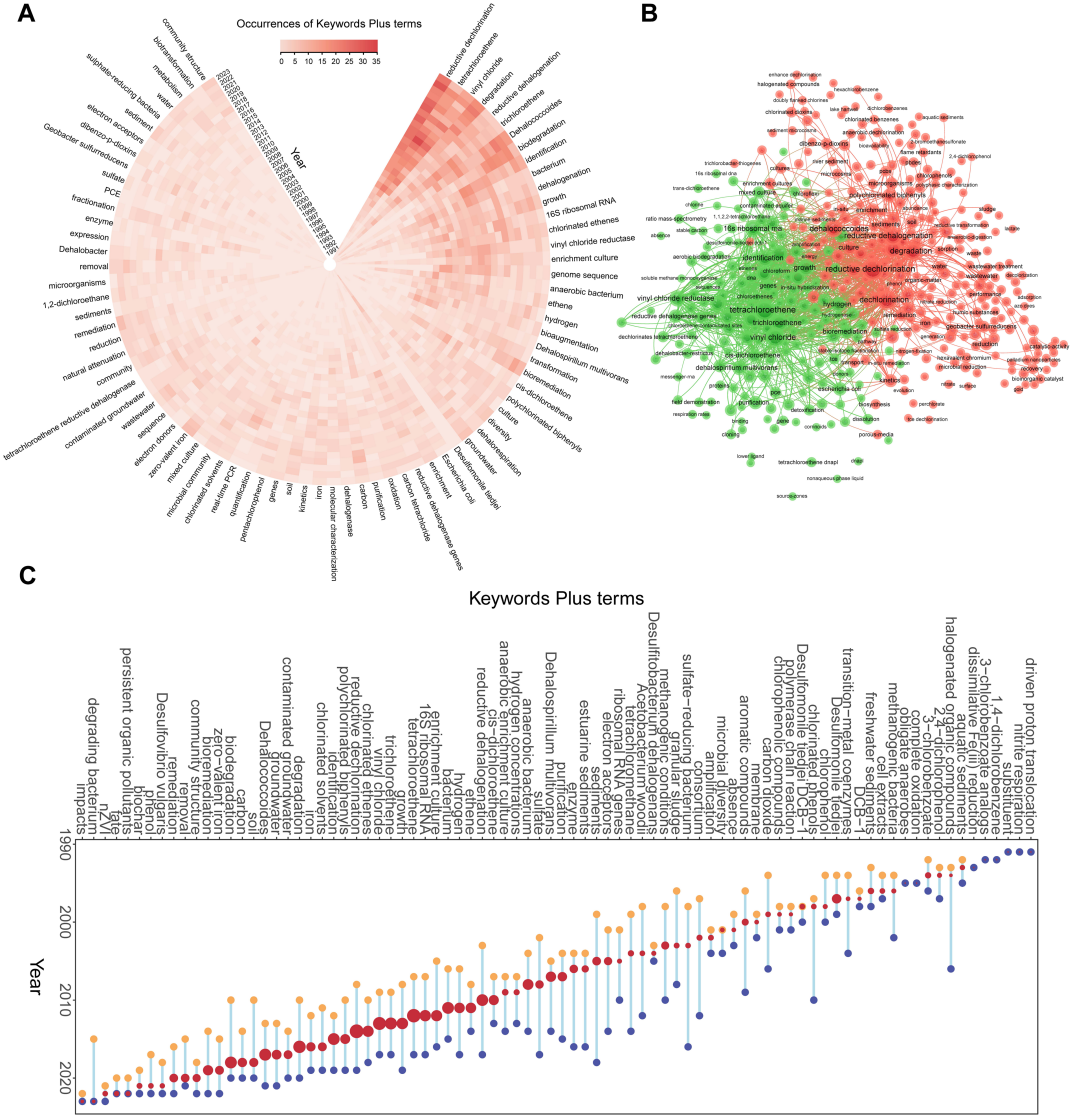
Research hotspots analysis. **(A)** Occurrences of Keywords Plus terms over time **(B)** Main Keywords Plus terms’ co-occurrence network. Each node represents one keyword, and the size of the nodes is positively correlated with the frequency of the keywords. Each color represents one cluster, and the thickness of the connecting lines between nodes is positively correlated with the co-occurrence frequency of the keywords. **(C)** Temporal trends in main Keywords Plus terms. The yellow, blue, and red dots, respectively, represent the first quarter, the third quarter, and median publication years for a given keyword. The size of the red dot signifies the number of publications. A red dot positioned further to the right and with a larger size indicates more recent publications and a greater research focus on the corresponding keyword (Xu et al., 2022).

Keywords serve as concise summaries of a publication’s core content, and their co-occurrence analysis offers valuable insights into research hotspots and emerging trends within a specific field (Pei et al., 2022; Xiang et al., 2017). Web of Science records encompass two types of keywords: Author keywords assigned by the original authors and Keywords Plus terms automatically extracted from reference titles (Zhang et al., 2016). This study opted to utilize Keywords Plus terms for analysis due to inherent limitations associated with Author keywords. Within this dataset, Author keywords demonstrated a comparatively low overall frequency and potential inconsistencies arising from factors such as synonym usage, abbreviations, and variations in spelling (Boudry et al., 2018). In contrast, Keywords Plus terms provide a more comprehensive and insightful perspective into the content, thereby bolstering the analytical rigor of the study (Garfield, 1990). Utilizing the “Words” function bar within the Bibliometrix package, an examination of Keywords Plus terms associated with OHRB research from 1988 to 2023 yielded a total of 2,633 distinct terms. High-frequency terms include “reductive dechlorination” (460 occurrences), “tetrachloroethene” (403 occurrences), “vinyl chloride” (307 occurrences), “degradation” (236 occurrences), “reductive dehalogenation” (221 occurrences), “trichloroethene” (190 occurrences), “*Dehalococcoides*” (187 occurrences), “biodegradation” (181 occurrences), and “identification” (177 occurrences). These terms have consistently dominated the research landscape (Figure 7A). Co-occurrence network analysis (Figure 7B) has delineated two principal thematic clusters within the study of organohalide respiration. The first cluster is anchored in the mechanisms of OHRB, encompassing terms such as “16S ribosomal RNA,” “genes,” “identification,” “purification,” “reductive dehalogenase genes,” “sequences,” and “proteins.” This focus underscores the importance of understanding the diversity, genetic and molecular mechanisms of OHRB, which are crucial for their identification and functional characterization. The second cluster highlights the interaction of OHRB with environmental factors, with terms like “hexavalent chromium,” “iron,” “water,” “soil,” “sediments,” “nitrate,” “wastewater,” “sludge,” “degradation,” and “perchlorate.” This cluster reflects the growing interest in how OHRB interact with and mitigate the effects of environmental pollutants, particularly in the context of composite pollution scenarios. The co-occurrence of “hexavalent chromium” in particular points to an increased emphasis on ecological restoration in environments affected by multiple pollutants and a growing interest in the interplay between organohalides and heavy metals. These clusters suggest that research in organohalide respiration is not only concerned with the fundamental biology and genetics of OHRB but also with their practical applications in environmental remediation. Temporal trends, visualized using Bibliometrix’s “Trend Topics” feature, indicate a shift towards applied research, with increasing emphasis on terms like “remediation,” “removal,” and “bioremediation” (Figure 7C). While terms like “corrinoids,” “corrinoid protein,” and “vitamin B_12_” appeared, their low frequency precluded inclusion in trend analysis. Overall, this keywords analysis effectively captures the evolving landscape of OHRB research, from foundational studies to applied environmental solutions.

## 4 Conclusion and perspectives

This study employed bibliometric methods and visualization tools to analyze selected literature on OHRB retrieved from the Web of Science Core Collection. The analysis revealed several key findings: (1) The overall quantity of publications related to OHRB research has been steadily increasing since 1988, and the mean total local citations per publication show a positive trend, indicating an increase in the average citation impact of OHRB research over time. (2) Environmental Science and Technology emerges as the journal with the highest publication quantity, while Applied and Environmental Microbiology maintains the most significant impact, although considerably less so in the past decade. (3) Leading authors with high number of publications showcase outstanding local impact. Their prolific scientific contributions have been concentrated in the past decade, reflecting close collaboration within their authorship groups. These findings highlight the collaborative nature of research in this field. (4) A clear regional disparity exists in OHRB research. The higher publication quantity, local impact, and international collaboration often occur in countries/regions in Europe and North America. While China has seen a recent surge in publications, its local impact and international collaboration remain relatively lower. This suggests potential areas for future collaboration and knowledge exchange. (5) Keywords analysis revealed terms like “reductive dechlorination,” “tetrachloroethene,” “vinyl chloride,” and “degradation” as frequently co-occurring, reflecting prominent research foci. Temporal trends in keywords suggest growing interest in areas like “remediation,” “removal,” “community structure,” “bioremediation,” and “zero-valent iron.” These evolving areas suggest potential new directions for OHRB research.

While existing research has provided a foundation for understanding dechlorination mechanisms and the capabilities of OHRB, significant knowledge gaps remain. These include a deeper understanding of electron transfer mechanisms, comprehensive characterization of key enzymes and proteins, and elucidation of the molecula mechanisms governing OHRB metabolism (e.g., enzyme kinetics and regulation, gene expression and regulation, and metabolic pathways and networks). Furthermore, optimizing the environmental applications of OHRB for *in situ* bioremediation requires translating laboratory findings to successful field-scale outcomes. This necessitates interdisciplinary collaboration between microbiologists, engineers, and environmental scientists to bridge the gap between fundamental research and practical implementation. Prioritizing these research areas will be crucial for advancing our understanding of these fascinating microorganisms and harnessing their full potential.

Based on our research experiences and the insights gained from this study’s identification of knowledge gaps, we propose several additional directions for future OHRB research. Importantly, our bibliometric analysis suggests that some of these areas remain under-explored, highlighting significant opportunities to expand the existing literature. While this perspective cannot comprehensively cover all unexplored aspects of OHRB, we aim to stimulate valuable discussions and further research in the field. We recommend prioritizing:

1. The role of OHRB in biogeochemical cycling. While we have identified a growing body of literature on OHRB-mediated reductive dehalogenation, our analysis revealed a relative lack of studies exploring the broader ecological implications of these processes for biogeochemical cycles. The investigation into the critical role of OHRB within biogeochemical element cycles represents a fundamental pursuit in microbial ecology and environmental science and holds profound significance for advancing our comprehension of environmental processes. OHRB, with their unique capacity to respire organohalides, emerge as key players in shaping the fate and transformation of halogenated compounds in diverse ecosystems and potentially exert a substantial influence on the cycling of biogeochemical elements (e.g., carbon, chlorine, bromine) (Leri and Myneni, 2010; Leri et al., 2015; Weigold et al., 2016). These bacteria not only influence the cycling of halogenated organic pollutants but also interact with various biotic and abiotic components of ecosystems, contributing to the overall dynamics of carbon, nitrogen, and other elements. Investigating the crucial role of OHRB in biogeochemical element cycles provides valuable insights into the interconnectedness of microbial life and geochemical processes, laying the foundation for informed environmental management and sustainable practices.
2. The complex interplay between OHRB and other microbial community members. Our keywords analysis has highlighted the popularity of studies focusing on individual OHRB strains or specific dehalogenation pathways. However, there is a need for more in-depth investigations into the ecological interactions between different OHRB species and their relationships with other microorganisms in the community. In recent years, unveiling the intricate interactions between different OHRB species and their relationships with community members has emerged as a pivotal research direction (e.g., resource competition, cross-feeding, growth inhibition, horizontal gene transfer) (Kruse et al., 2021; Lipson et al., 2021; Maphosa et al., 2012; Wang et al., 2024; Yuan et al., 2021). This inquiry delves into the dynamic ecological interplay within microbial communities, shedding light on how OHRB coexist and compete with other microorganisms (e.g., nitrate-reducing bacteria, sulfate-reducing bacteria, iron-reducing bacteria, methanogens, acetogens, fermenters) in complex environments. Investigating resource competition provides insights into the factors driving OHRB adaptability and survival strategies. The exploration of cross-feeding dynamics (e.g., cobamide exchange) elucidates the cooperative relationships that may enhance the efficiency of organohalide turnover and biodegradation. Understanding growth inhibition mechanisms contributes to deciphering the ecological balance within microbial consortia. Furthermore, unraveling horizontal gene transfer events unveils potential mechanisms for the dissemination of key genetic traits among microbial communities. This comprehensive investigation has implications not only for fundamental microbial ecology but also for optimizing OHRB-mediated bioremediation strategies by harnessing the synergies or mitigating the challenges posed by these intricate microbial interactions.
3. The evolutionary history of OHRB. A crucial frontier in understanding OHRB lies in exploring their evolutionary history and the processes that shaped their unique metabolic capabilities. While recent studies have begun to investigate this area (Yang et al., 2020b; Yang et al., 2022), a deeper examination into the temporal and spatial dynamics of OHRB evolution is needed. Understanding the evolutionary history of OHRB is essential for unraveling the genetic and adaptive mechanisms that have shaped their unique ability to respire organohalides. Such advancements will enable the identification of key genetic determinants, regulatory networks, and ecological drivers responsible for the emergence, persistence, and diversification of OHRB across diverse environments. Ultimately, such insights will not only expand our fundamental knowledge of these specialized anaerobes but also illuminate origins and strategies for microbial energy conservation.
4. The application of synthetic biology and genetic manipulation. While some studies have explored the genetic engineering of OHRB, our analysis suggests that this area remains relatively under-explored. Synthetic biology and genetic manipulation represent crucial strategies for advancing OHRB research, enabling the development of more efficient and adaptable strains for diverse applications (Grostern et al., 2009; Halliwell et al., 2021; Kunze et al., 2017; Mac Nelly et al., 2014; Molenda et al., 2019; Picott et al., 2022). This domain focuses on deploying cutting-edge technologies to engineer OHRB and OHRB microbial communities with precision and finesse. Central to this endeavor is the development of OHRB chassis, incorporating synthetic genes and other genetic elements. By employing synthetic biology techniques, researchers aim to construct tailored OHRB or assembly of OHRB communities that exhibit enhanced capabilities for organohalide biotransformation. The targeted genetic manipulation enables the optimization of OHRB and microbial consortia, offering potential breakthroughs in understanding the functions of characterized genes as well as the efficiency and specificity of organohalide biotransformation processes. For instance, exploring the potential of genetic engineering to develop reductive dehalogenases (RDases) capable of breaking carbon-fluorine (C-F) bonds holds significant promise for the remediation of per- and polyfluoroalkyl substances (PFAS) (Hu and Scott, 2024). This approach not only provides a platform for technological innovation but also promises to deepen our understanding of the intricate genetic factors governing OHRB behavior, fostering the development of sustainable and effective strategies for environmental remediation.
5. The dehalogenation potential of archaea. Our analysis identified a significant knowledge gap concerning the role of archaea in dehalogenation processes. Investigating archaeal dehalogenation capabilities stands out as a critical direction for future research within the field of organohalide-respiring microorganisms. While much attention has been dedicated to understanding the mechanisms employed by bacterial counterparts, the role and potential contributions of archaea in dehalogenation processes remain relatively under-explored. Recent studies employing omics approaches have provided evidence that certain archaeal genomes, including draft genomes, harbor genes encoding reductive dehalogenases (Firrincieli et al., 2021; Greening et al., 2024; Han et al., 2024; Krzmarzick et al., 2018; Manoharan et al., 2019; Spang et al., 2019; Zhang et al., 2021). These findings suggest that archaea may possess the capability to harvest energy by utilizing halogenated compounds as terminal electron acceptors, potentially contributing to the natural turnover of halogenated compounds. Investigating the dehalogenation capabilities of archaea presents an opportunity to broaden our comprehension of microbial-driven organohalide transformations. This line of inquiry could unveil unique enzymatic pathways, metabolic strategies, and ecological implications associated with archaeal-mediated dehalogenation. Such insights could not only enhance our understanding of the intricate microbial interactions within pristine or contaminated environments but also pave the way for innovative approaches in harnessing archaeal dehalogenation capabilities for effective bioremediation strategies and environmental management.

## 5 Limitations

This bibliometric analysis offers a comprehensive overview of publications within the OHRB field from 1988 to 2023. The findings provide valuable insights into the current research status, hotspots, and development trends in the field of OHRB research. However, it is crucial to acknowledge the limitations associated with data acquisition and analysis.

1. Publication bias: In order to ensure the scientific rigor and authority of the data and conclusions of this study, as well as to meet the requirements of bibliometric tools regarding data types, the WoSCC database was selected as the data source for this research. However, the study relies exclusively on documents sourced from the WoSCC database, which means it may omit relevant contributions from other repositories and sources.
2. Screening bias: Due to the predominance of English language publications in the retrieval of OHRB-related literature, this study has set the language type as English and the type of documents as research and review articles as the selection criteria for publication retrieval, aiming to present the research results more effectively (You et al., 2021). Additionally, to ensure the relevance of the included literature, this study defines OHRB-related terms as the research theme of the WoSCC database. For certain publications, even though OHRB is mentioned in their text, if it is not classified by the retrieval system as having OHRB as the main topic, they will still be excluded. In the initial stage of OHRB research, some publications may also be excluded from the search system due to the lack of a clear definition of OHRB. Furthermore, due to the diversity of search terms, it is inevitable that some relevant publications may have been missed. Despite not being captured in this analysis, the enduring impact of some influential studies cannot be ignored. The setup of these selection criteria listed in the method section may introduce a certain degree of screening bias.
3. Algorithm bias: The main conclusions of this study depend on the utilization of two bibliometric tools, namely the Bibliometric software package and VOSviewer. Although efforts have been made to manually verify and clean the relevant data to enhance the accuracy of the analyses, machine algorithms often fall short compared to human intelligence in handling analysis (Ma et al., 2020). While machine algorithms were utilized, the authors have meticulously verified relevant data to ensure the accuracy of the analyses and data presented, minimizing the potential for errors introduced by automated processes.

## Data Availability

The original contributions presented in the study are included in the article/Supplementary material, further inquiries can be directed to the corresponding author/s.

## Footnotes

^1^ https://serdp-estcp.mil

^2^ https://gepris-extern.dfg.de/gepris/projekt/171475307
