# Supplementary material for: Organohalide respiration: retrospective and perspective through bibliometrics
Source: Front Microbiol. 2024 Dec 24;15:1490849. doi: 10.3389/fmicb.2024.1490849 (PMC11703978; doi:10.3389/fmicb.2024.1490849)
Supplement: Supplementary file 1 [file Table_1.docx]

Supplementary Table S1 Cumulative number of publications (CNP) and annual number of publications (ANP) over time.

| Year | ANP | CNP |
| --- | --- | --- |
| 1988 | 1 | 1 |
| 1989 | 1 | 2 |
| 1990 | 1 | 3 |
| 1991 | 6 | 9 |
| 1992 | 8 | 17 |
| 1993 | 5 | 22 |
| 1994 | 11 | 33 |
| 1995 | 8 | 41 |
| 1996 | 11 | 52 |
| 1997 | 16 | 68 |
| 1998 | 15 | 83 |
| 1999 | 20 | 103 |
| 2000 | 15 | 118 |
| 2001 | 18 | 136 |
| 2002 | 23 | 159 |
| 2003 | 30 | 189 |
| 2004 | 44 | 233 |
| 2005 | 33 | 266 |
| 2006 | 51 | 317 |
| 2007 | 52 | 369 |
| 2008 | 57 | 426 |
| 2009 | 50 | 476 |
| 2010 | 58 | 534 |
| 2011 | 66 | 600 |
| 2012 | 67 | 667 |
| 2013 | 83 | 750 |
| 2014 | 84 | 834 |
| 2015 | 74 | 908 |
| 2016 | 83 | 991 |
| 2017 | 88 | 1079 |
| 2018 | 84 | 1163 |
| 2019 | 85 | 1248 |
| 2020 | 87 | 1335 |
| 2021 | 83 | 1418 |
| 2022 | 97 | 1515 |
| 2023 | 76 | 1591 |
